# Highly Thermally Stable and Miscible CO_2_‑Based Block Copolymers by the Combination of Ring-Opening and RAFT Copolymerizations through Mediated Hydrogen Bonding Interactions

**DOI:** 10.1021/acs.macromol.5c03069

**Published:** 2026-01-26

**Authors:** Yen-Ling Kuan, Yu-Chun Chiu, Yun-Sheng Ye, Shiao-Wei Kuo

**Affiliations:** Department of Materials and Optoelectronic Science, Center for Functional Polymers and Supramolecular Materials, National Sun Yat-Sen University, Kaohsiung 80424, Taiwan

## Abstract

In this study, the chain end of a reversible addition–fragmentation chain transfer (RAFT) polymerization agent of poly­(cyclohexene carbonate) (PCHC) was synthesized via the ring-opening copolymerization of CO_2_ and cyclohexene oxide (CHO) by using s-dodecyl-s’-(α,α′-dimethyl-α″-acetic acid) trithiocarbonate (DDMAT) as a chain transfer agent. Various block copolymers of poly­(cyclohexene carbonate)-*b*-poly­(styrene-*alt-N*-(hydroxyphenyl)­maleimide) (PCHC-*b*-PSHPMI) were subsequently synthesized by the RAFT copolymerization of styrene and *N*-(hydroxyphenyl)­maleimide (HPMI) in the presence of azobis­(isobutyronitrile) (AIBN), which were characterized by using differential scanning calorimetry (DSC), thermogravimetric analysis (TGA), Fourier transform infrared (FTIR) spectroscopy, nuclear magnetic resonance (NMR), and gel permeation chromatography (GPC). DSC thermal analyses indicated that the single *T*_g_ values were observed for all PCHC-*b*-PSHPMI copolymers, indicating miscible behavior, and the *T*_g_ value was 194 °C for the PCHC-*b*-PSHPMI78 copolymer. One- and two-dimensional (2D) FTIR spectroscopy revealed that these PCHC-*b*-PSHPMI copolymers actually provide relatively weak intermolecular O–H···O=C hydrogen bonding, which was attenuated by the self-association of hydrogen bonding within the pure PCHC and pure PSHPMI segments. In the solid-state ^13^C NMR spectra, a pronounced chemical shift variation of the C–OH and C=O units of the PSHPMI segment and C=O units of the PCHC segment was also observed, which is attributable to the intermolecular hydrogen interactions in these PCHC-*b*-PSHPMI copolymers. Rotating-frame ^1^H spin–lattice relaxation [*T*_1ρ_(H)] analyses also indicated the complete miscible behavior of these block copolymers within the 2–3 nm length scale, and the relaxation times exhibited positive deviations from the linear predicted rule. These results suggest that the loose chain structure was formed because of the weaker intermolecular hydrogen bonding between the PCHC and PSHPMI segments in the block copolymers.

## Graphical abstract

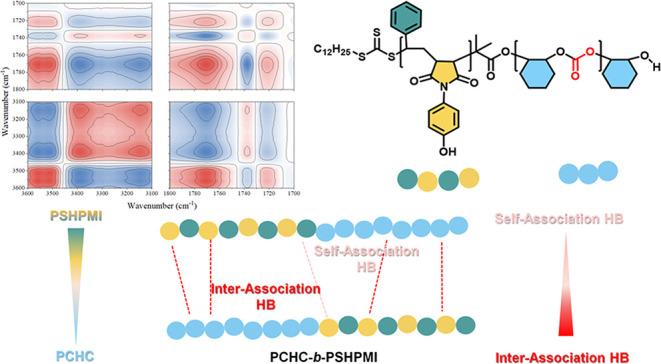

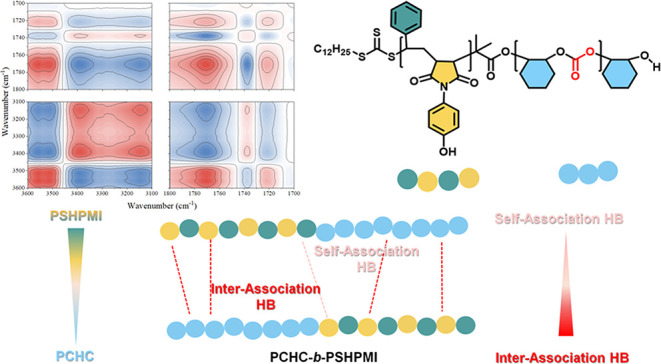

## Introduction

Carbon capture and utilization (CCU) provides a range of applications that capture carbon dioxide (CO_2_) from sources such as industrial facilities and then reuse it directly, either directly or indirectly, to create new products such as CO_2_-based synthetic fuels, chemicals, and polymeric materials.^1–3^ CO_2_ utilization is a key process for reducing carbon emissions, and copolymerization with carbon-negative chemicals could lower the atmospheric CO_2_ levels significantly.^4–6^ The green reactions of epoxides such as propylene oxide (PO) or cyclohexene oxide (CHO) with CO_2_ could produce polycarbonates (PPC or PCHC).^7–10^

The relatively low glass transition temperature (*T*_g_) with poor mechanical and dimensional stability of PPC (*T*_g_ = 30 °C) and PCHC (*T*_g_ = 108 °C) also exhibit intrinsic brittleness.^11,12^ Because of the presence of C=O units of these CO_2_-based copolymers, the blending with polar functional polymers with O–H or C=O units have been selected to enhance their thermal and mechanical properties such as those of cellulose, ethylene-*co*-vinyl alcohol (EVOH) copolymer, starch, poly­(methyl methacrylate) (PMMA), and poly­(lactic acid) (PLA)^13–18^ through hydrogen bonding or dipole–dipole interactions as expected. However, these studies revealed that PPC or PCHC intrinsically exhibits intramolecular hydrogen bonding, thereby diminishing the strength of the intermolecular interactions of most binary blend systems.^12,19^ For example, we blended a typical strong hydrogen-bonded polymer, poly­(vinylphenol) (PVPh, *T*_g_ = 168 °C), with PCHC, which resulted in a miscible binary blend; however, due to the relatively weak intermolecular hydrogen bonding in PVPh/PCHC binary blends, a negative deviation in *T*_g_ behavior was observed based on the Fox and Kwei equation.^12^ For example, the PVPh/PCHC = 50/50 binary blend exhibited a single *T*_g_ value of 107 °C, even lower than that of pure PCHC, and only the PVPh/PCHC = 70/30 and 80/20 blends displayed relatively higher *T*_g_ values of 136 and 140 °C, respectively. The main reason is that the PCHC copolymer intrinsically exhibits intramolecular hydrogen bonding, thereby diminishing the strength of the intermolecular interactions upon blending with PVPh and resulting in a negative deviation based on the linear rule from the *T*_g_ behavior.^12^

Subsequent terpolymerization, in which a third comonomer, such as an anhydride or other epoxide derivative, was introduced to increase the strength of the hydrogen-bond acceptor sites, still yielded a negative deviation in the *T*_g_ behavior.^19–25^ To address the limitations of binary blend systems, CO_2_-based copolymers were designed in this study through covalent-linked hydrogen-bonded donor segments to form block copolymers. This is because of the intramolecular screening and functional group accessibility that result in the difference in the degree of rotational freedom between polymer blends and block copolymers.^26–29^ Controlled radical copolymerization (CRP), such as reversible addition–fragmentation chain transfer (RAFT) polymerization,^30–33^ was used to prepare the PCHC-based block copolymer through a covalent-linked hydrogen-bonded donor segment, as it can enable the precise control over the segment length of hydrogen-bonded donor monomers.^34–39^

However, the vinylphenol monomer generally could not be copolymerized directly by using conventional free radical copolymerization because of the side reactions and chain transfer processes that result in the low molecular weight of the PVPh segment.^40^ The protective functional groups, such as acetoxystyrene (AS) or butoxystyrene (BOS), could be synthesized by free radical polymerization;^41–44^ however, the subsequent hydrolysis reaction to form PVPh segments in HCl or NaOH media may also break the carbonate or ether units in CO_2_-based copolymer segments. As a result, to circumvent this limitation, *N*-(hydroxyphenyl)­maleimide (HPMI) monomer bearing phenolic OH unit was selected directly as a source of hydrogen-bonded donor segment, which is widely used in compounding formulations of phenolic resins to provide good thermal properties.^45^ The alternating copolymerization of styrene and HPMI to form poly­(S-*alt*-HPMI) copolymers generally results in good thermal properties (*T*_g_ > 250 °C) and chemical stability,^40,45^ which have been widely discussed in our previous studies.^46–49^ In these studies, the phenolic OH units of the HPMI segments serve as hydrogen-bonded donor sites, while the styrene units in the alternating sequence act to effectively dilute the donor moieties, thereby mitigating the excessive self-association of HPMI and modulating the overall intermolecular interactions.^40^

In this study, *s*-dodecyl-*s’*-(α,α′-dimethyl-α″-acetic acid) trithiocarbonate (DDMAT) was employed as a chain transfer agent for the copolymerization of cyclohexene oxide (CHO) with CO_2_ to yield the PCHC–DMMAT chain transfer agent. Subsequent RAFT-mediated copolymerization of styrene with HPMI afforded PCHC-*b*-PSHPMI block copolymers. Owing to the precise polymerization control provided by the RAFT process, a series of block copolymers with systematically varied PSHPMI compositions or molecular weights were synthesized. The influence of hydrogen-bonded donor composition on the thermal properties, miscibility, hydrogen-bonding interactions, and domain size of these block copolymers was then systematically investigated by using DSC, FTIR, and NMR analyses in this study. For comparison, PCHC/PSHPMI binary blends were also prepared, and their thermal behavior was analyzed by using DSC thermal analyses.

## Experimental Section

### Materials

Sodium hydroxide (97%) and azobis­(isobutyronitrile) (AIBN, 99%) were purchased from SHOWA. 1-Dodecanethiol (98%) was purchased from Thermo Scientific. Hydrochloric acid (HCl), calcium hydride (CaH_2_), and Aliquat (R) 336 TG were purchased from Alfa-Aesar. Carbon disulfide (99.9%) was purchased from Honeywell. Hydrochloric acid (35%) was purchased from Aencore. Chloroform (99.8%) was purchased from Fisher Chemicals. Acetone (99.5%) was purchased from Seedchem. Cyclohexene oxide (CHO, 98%), styrene (99%), dimethylformamide (DMF), and tetrahydrofuran (THF) were purchased from Acros. *N*-hydroxyphenylmaleimide (HPMI, 98%) was purchased from Aladdin. High-purity carbon dioxide (CO_2_, >99.999%) was purchased from Hsin E-Li Co., Ltd. Before use, CHO, DMF, and THF were refluxed with CaH_2_ for half a day and vacuum-distilled. The chemical structure and the synthetic catalyst of LZn_2_(OAc)_2_ are summarized in Schemes S1 and S2.^12^ The synthesis and chemical structure of the chain transfer agent of s-dodecyl-s’-(α,α′-dimethyl-α″-acetic acid) trithiocarbonate (DDMAT) are summarized in Figure S1 and Scheme S3.^50^

### Copolymerization of Poly­(cyclohexene carbonate) with DDMAT (PCHC-DDMAT)

CHO (16 mL, 0.158 mol) was introduced into a round-bottom flask to dissolve DDMAT (0.97 g, 2.67 mmol). LZn_2_(OAc)_2_ (0.16 g, 0.267 mmol) was dried under vacuum at 100 °C for 3 h in an autoclave. Once cooled, the autoclave was purged with CO_2_, and the CHO and DDMAT mixture was introduced. The copolymerization was carried out in an oil bath at 80 °C for 20 h under a constant CO_2_ pressure of 435 psi. After completion, the reactor was cooled, and the residual CO_2_ was carefully vented. The mixture was dissolved in dichloromethane and extracted with 5 wt % HCl. The product was then precipitated twice using methanol. FTIR (KBr, cm^–1^): 1750 (C=O); ^1^H NMR (500 MHz, CDCl_3_, δ, ppm): 0.88 (t, 3H, −CH_2_C**H**_**3**_), 1.25–1.29 (s, 8H, CyC**H**_2_), 1.71 (m, 2H, −C**H**_**2**_CH_2_S−),1.74 (s, 6H, −C–C**H**_**3**_), 3.19 (t, 2H, −C**H**_**2**_S−), 4.40–4.82 (s, 1H, CyC**H**OCO_2_).

### Synthesis of PCHC-*b*-P­(S-*alt*-HPMI) Block Copolymers by RAFT

PCHC-DDMAT (0.5 g, 0.7 mmol), AIBN (0.00233 g, 0.014 mmol), styrene (0.17 g, 0.0034 mol), HPMI (0.645 g, 0.0034 mol), and 5 mL of DMF were charged into a dried round-bottom flask, which was degassed by three freeze–pump–thaw cycles, sealed, and heated for 24 h at 80 °C. The mixture was cooled to room temperature and reprecipitated with methanol. Block copolymers with other compositions were synthesized using the same procedure and designated as PCHC-*b*-PSHPMI17, PCHC-*b*-PSHPMI29, PCHC-*b*-PSHPMI51, PCHC-*b*-PSHPMI72, and PCHC-*b*-PSHPMI78, corresponding to the weight percentages of the P­(S-*alt*-HPMI) segment in each copolymer. FTIR (KBr, cm^–1^): 1750 and 1704 (C=O); ^1^H NMR (500 MHz, CDCl_3_, δ, ppm): 0.88 (t, 3H, −CH_2_C**H**_**3**_), 1.25–1.29 (s, 8H, CyC**H**_2_), 1.71 (m, 2H, −C**H**_**2**_CH_2_S−),1.74 (s, 6H, −C–C**H**_**3**_), 4.40–4.82 (s, 1H, CyC**H**OCO_2_), 6.77 (m, 2H, Ar**H**), 7.17 (m, 2H, Ar**H**), 7.11 (s, 2H, Ar**H**), 9.72 (s, 1H, O**H**).

## Results and Discussion

### Synthesis of the PCHC-DDMAT Copolymer

As depicted in Figure 1a, the PCHC-DDMAT copolymer was synthesized via the ring-opening copolymerization of cyclohexene oxide (CHO) and CO_2_, employing LZn_2_(OAc)_2_ as the catalyst and DDMAT as the chain transfer agent. Williams and co-workers reported highly active binuclear and polynuclear zinc catalysts, including salen-based complexes, for the copolymerization of CO_2_ and epoxides, achieving efficient polycarbonate formation at low catalyst loadings.^4^ Accordingly, salen Zn catalysts were selected based on prior literature, with the dinuclear zinc catalyst LZn_2_(OAc)_2_ specifically chosen for this CO_2_ polymerization; it promotes the formation of alternating copolymers from CHO and CO_2_ while minimizing polyether linkages, as consistently demonstrated in established studies. The FTIR spectrum in Figure 1b displays the absorption peak of C=O at ca. 1750 cm^–1^, corresponding to the carbonate group of the PCHC segment. Figure 1c shows the ^1^H NMR spectrum of the PCHC-DDMAT copolymer, where the characteristic signals for the cyclohexyl CH unit of PCHC on the main chain appear at 4.6 ppm, and the CH_2_ signal of DDMAT appears at 3.2 ppm. Additionally, the repeat unit of PCHC was calculated from the signals of PCHC and DDMAT using the integral area of ^1^H NMR spectroscopy, and the data are displayed in Table 1. Therefore, the conversion and selectivity of PCHC-DDMAT are shown in Table S1, with a selectivity of 98%. Figure 1d shows the ^13^C NMR spectrum, featuring the carbon signals for the C=O of the carbonate group at 154 ppm, and the signal was observed at ca. 22–29 ppm for aliphatic CH_2_ carbons of the DDMAT segment.

**Figure 1:**
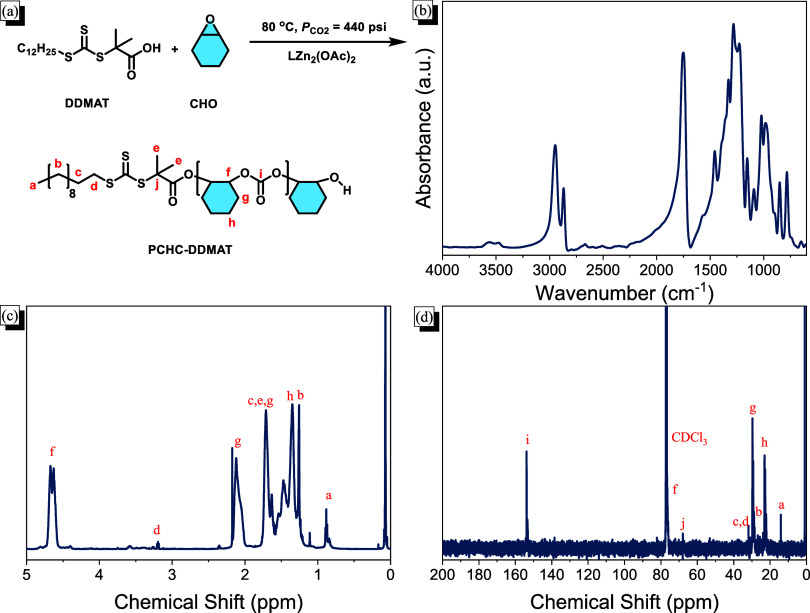
(a) Chemical structure and the synthesis of the PCHC-DDMAT copolymer and its corresponding (b) FTIR, (c) ^1^H, and (d) ^13^C NMR spectra.

**Table 1:** Characteristics of Various PCHC-b-PSHPMI Block Copolymers Synthesized in This Study

	SHPMI (wt %)^a^			^a^	^b^	^b^	^c^	^c^
	monomer feed	polymer composition	*T*_g_ (°C)	*M*_n_ (NMR)^a^	*M* _n_ (GPC)^b^	*Đ* ^b^	*T*_d10_ (°C)^c^	*T*_360 °C_ (wt %)^c^
PCHC-DDMAT	0	0	108	5000	5700	1.34	312	0
PCHC-*b*-PSHPMI17	15.7	17.0	100	5900	6900	1.29	303	21
PCHC-*b*-PSHPMI29	27.3	29.0	103	6800	8600	1.23	300	33
PCHC-*b*-PSHPMI51	49.5	51.0	115	14,000	11,100	1.19	297	53
PCHC-*b*-PSHPMI72	66.7	72.6	176	24,000	14,400	1.22	300	73
PCHC-*b*-PSHPMI78	75.0	78.6	194	31,500	14,900	1.18	298	75
PSHPMI^d^	100	100	250		18,800	1.57	409	100

a Measured by ^1^H NMR.

b Measured by GPC.

c Measured by TGA.

d Synthesis by free radical copolymerization (Scheme S4).

### Characterization of PCHC-*b*-PSHPMI Block Copolymers by RAFT

The synthesis of PCHC-*b*-Poly­(S-*alt*-HPMI) (PCHC-*b*-PSHPMI) block copolymers was conducted via RAFT polymerization of styrene and HPMI monomers with PCHC-DDMAT as a macroinitiator, as shown in Figure 2a, which was investigated by FTIR, ^1^H, and ^13^C NMR spectra. The block copolymers were prepared using various ratios of styrene/HMPI (1/1 molar ratio) with PCHC-DDMAT. The characteristic C=O signals of the carbonate and anhydride units appear at 1750 and 1704 cm^–1^ in the FTIR spectra, respectively, as shown in Figure 2b. The intensity of the C=O signals at 1704 cm^–1^ of PSHPMI increased with increasing PSHPMI composition. In addition, the OH stretching signal also exhibited the same trend as the C=O unit, as would be expected.

**Figure 2:**
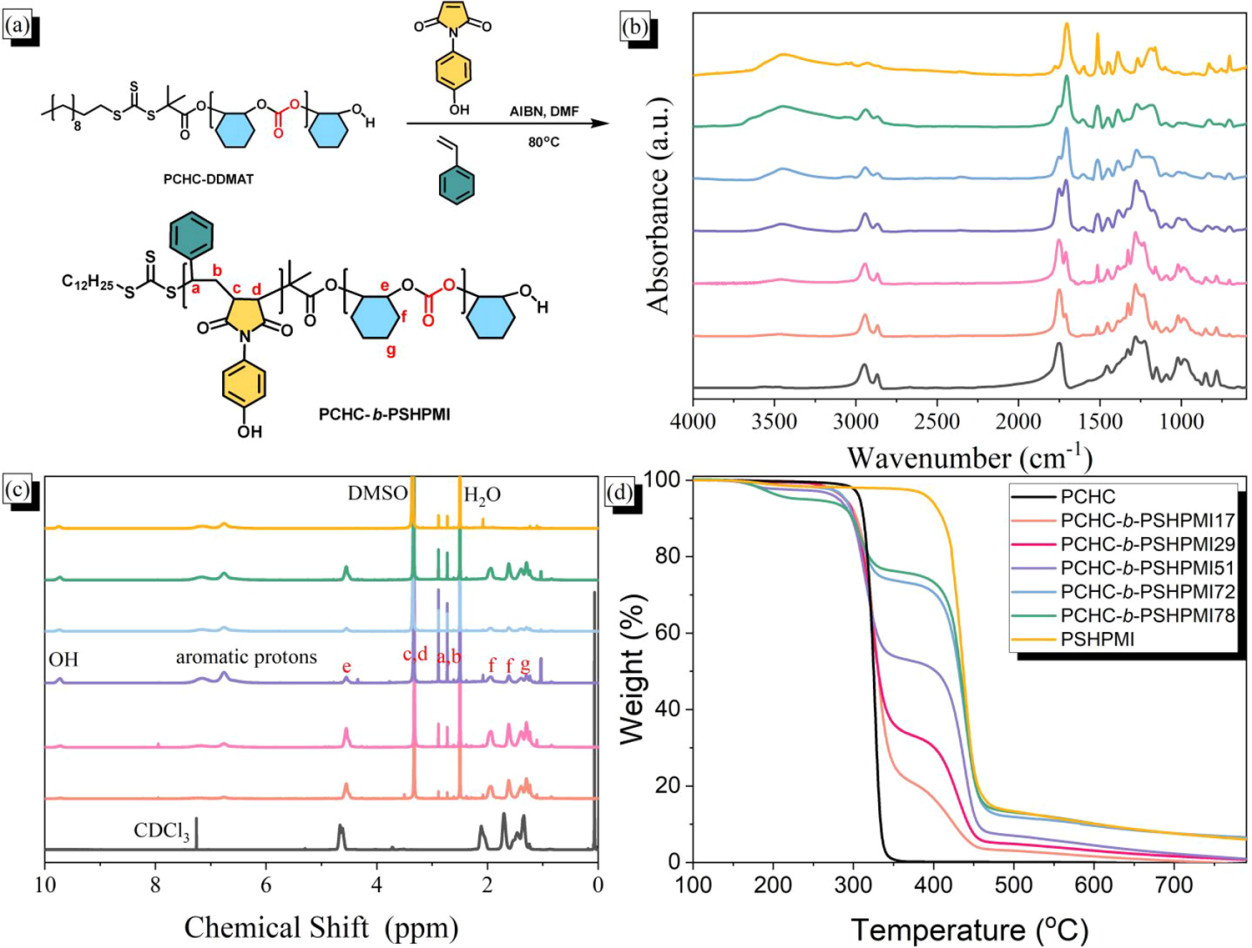
PCHC-*b*-PSHPMI diblock copolymers: (a) chemical structure and the synthesis route, (b) FTIR, (c) ^1^H NMR spectra, and (d) TGA analyses. The block copolymer compositions (b) and (c) are the same as in (d).

In the ^1^H NMR spectra of the pure poly­(S-*alt*-HPMI) alternating copolymer, as displayed in Figure 2c, signals appeared for the aromatic protons at 6.6–7.4 ppm and for the phenolic OH units at 9.7 ppm. The cyclohexyl CH signal, aromatic protons, and phenolic OH groups appeared simultaneously after block copolymerization. The integral area ratios from the PCHC and PSHPMI segments could be used to calculate the polymer compositions of the PCHC-*b*-PSHPMI copolymers based on cyclohexyl CH (4.6 m) and aromatic protons (6.6–7.4 ppm), as illustrated in Table 1. Figure S2 shows the corresponding ^13^C NMR spectra, where the signals appear for C=O at 179.0 ppm, for C–OH at 157.8 ppm, and for aromatic carbon at 115.4, 128.1, and 128.6 ppm. From the PSHPMI block segment, the signals appear for C=O at 154.2 ppm from the PCHC block segment, also indicating the successful synthesis of PCHC-*b*-PSHPMI copolymers. In the TGA analyses, the pure PCHC and PSHPMI showed one-step degradation, whereas the PCHC-*b*-PSHPMI block copolymers showed two-step degradation profiles. The first stage of weight loss was due to the PCHC segment, and the second stage corresponded to the PSHPMI segment, as expected, as displayed in Figure 2d. When the temperature reached 360 °C, the yields of the char were 21, 33, 53, 73, and 75 wt %, which are close to the PSHPMI composition in the block copolymer, as determined by ^1^H NMR analyses (Table 1). Furthermore, the *T*_d10_ value of the PCHC-*b*-PSHPMI block copolymer was lower than that of the pure PCHC and PSHPMI homopolymers. This reduction is attributed to the dodecyl trithiocarbonate end groups, which are known to destabilize the onset of thermal degradation, consistent with prior literature reports.^51^

Figure 3a shows the GPC analyses of various PCHC-*b*-PSHPMI block copolymers. The molecular weight (decrease of retention time with monodistribution) of PCHC-*b*-PSHPMI increased as the PSHMPI composition increased, indicating the formation of block copolymers. The successful synthesis of PCHC-*b*-PSHPMI was demonstrated by DOSY ^1^H NMR spectroscopy, which displayed a single diffusion coefficient, as shown in Figure 3b. As a result, the successful synthesis of the PCHC-*b*-PSHPMI copolymer was confirmed by using FTIR, NMR, and GPC analyses in this study. Furthermore, at higher PSHPMI composition, the PSHPMI block exhibits strong self-association and intrachain hydrogen bonding, as well as π–π stacking between the aromatic units. These interactions induce a compact, folded chain conformation in solution rather than an expanded random-coil geometry, and such compact conformations result in a substantially smaller apparent hydrodynamic radius, leading to a systematic underestimation of the molecular weight by GPC analyses.

**Figure 3:**
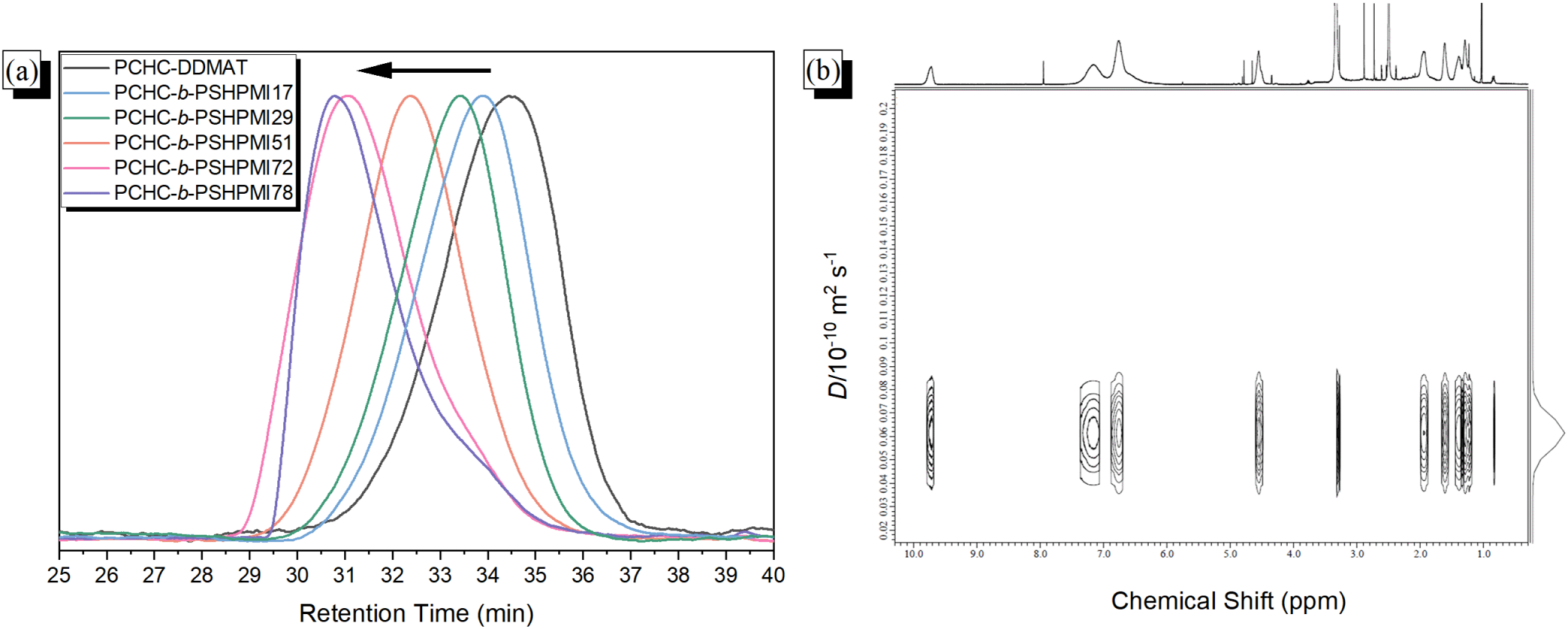
(a) GPC analyses of various PCHC-*b*-PSHPMI block copolymers, and (b) DOSY ^1^H NMR spectra of the PCHC-*b*-PSHPMI51 block copolymer.

### Thermal Analyses and Hydrogen-Bonding Interactions of PCHC-*b*-PSHPMI Block Copolymers

Figure 4A presents the results of DSC thermal analyses for pure PCHC, pure PSHPMI, and various PCHC-*b*-PSHPMI block copolymers. Each block copolymer composition exhibited a single *T*_g_ value, indicating that an intermolecular hydrogen-bonding interaction existed between the PCHC and PSHPMI block segments, which led to miscibility between the two block segments. The pure PCHC and PSHPMI segments display single *T*_g_ values at 108 and 250 °C, respectively. Clearly, at lower PSHPMI composition (<51 wt %), the *T*_g_ value is similar to that of the pure PCHC segment (*T*_g_ = 103–115 °C), which is significantly lower than the predicted value by the Fox equation and comparable to that of blending PVPh homopolymer.^12^ The strong intramolecular hydrogen bonding of the PCHC segment limits the interassociation interactions with other polymers. However, at relatively higher PSHPMI compositions (72 and 78 wt %), the *T*_g_ values were 176 and 194 °C for PCHC-*b*-PSHPMI72 and PCHC-*b*-PSHPMI78 copolymers, respectively, which could also be predicted using the Fox equation, indicating that the self-association hydrogen-bonding strength of the PSHPMI segment is similar to the interassociation hydrogen-bonding strength between the PCHC and PSHPMI segments at these compositions. In addition, to the best of our knowledge, the *T*_g_ value of 194 °C is the highest *T*_g_ value for a CO_2_-based copolymer system. In general, the modified Kwei equation is appropriate for characterizing the *T*_g_ values of miscible blends or copolymers with hydrogen bonding in order to better explain the *T*_g_ behavior of these blends or copolymers:^52,53^

1 $$T_{g}=\frac{W_{1}T_{g1}+{\mathrm{kW}}_{2}T_{g2}}{W_{1}+{\mathrm{kW}}_{2}}+qW_{1}^{2-\alpha }W_{2}^{\alpha }$$

**Figure 4:**
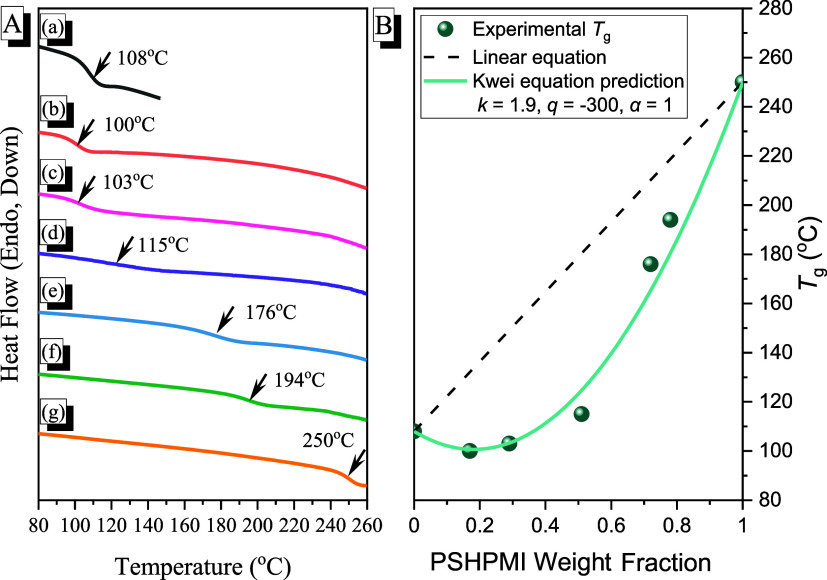
(A) DSC thermal analyses of (a) PCHC, (b) PCHC-*b*-PSHPMI17, (c) PCHC-*b*-PSHPMI29, (d) PCHC-*b*-PSHPMI51, (e) PCHC-*b*-PSHPMI72, (f) PCHC-*b*-PSHPMI78, and (g) PSHPMI. (B) Corresponding *T*_g_ values predicted by the linear rule and the Kwei equation.

where *T*_g1_ and *T*_g2_ are the *T*_g_ values of the corresponding PCHC and PSHPMI block segments, respectively, and *W*_1_ and *W*_2_ are the weight fractions of each block copolymer composition. The constants in the model are fitted by the parameters *k* and *q*. In particular, *q* represents the specific hydrogen-bonding interactions between copolymer segments, and *k* is the ratio of the volume expansion coefficients of the pure copolymer segments. However, the self-association of the hydrogen-bonded donor groups can also have an impact on the polymer blends or copolymers, as hydrogen bonding in polymer blends or copolymers is not just confined to the interassociation of hydrogen-bonding donors and acceptors. By incorporation of α, the model offers a more thorough explanation of the intermolecular hydrogen bonding found in these polymer blends or copolymers, improving its capacity to forecast their thermal and physical characteristics. The linear equation did not fit the PCHC-*b*-PSHPMI copolymers; however, the modified Kwei equation can fit all of the *T*_g_ values of the PCHC-*b*-PSHPMI copolymer (*k* = 1.9, *q* = −300, α = 1), as shown in Figure 4B.

The *T*_g_ behavior in hydrogen-bonded polymer blends or block copolymers is well known to deviate from simple mixing rules, particularly when both components exhibit strong self-association. In such systems, the competition between self-association and interassociation hydrogen bonding often leads to pronounced nonideal *T*_g_ behavior rather than a Fox-type dependence.^54^ Indeed, strongly negative or plateau-type *T*_g_ deviations have been widely reported in hydrogen-bonded polymer systems, including phenolic/phenoxy blends,^55^ PVPh/phenoxy blends,^56^ and PVPh/phenolic blends^57^ or weak hydrogen bonding in PAS/PEO binary blends,^58^ all of which exhibit behavior highly analogous to the present PCHC-*b*-PSHPMI copolymers. At low PSHPMI contents (≤50 wt %), *T*_g_ remains close to that of PCHC because the PCHC segments form strong intrachain and interchain self-associated hydrogen bonds that dominate the segmental dynamics. Although interassociation hydrogen bonding between the PCHC carbonyl groups and the PSHPMI OH groups is present, it is insufficient to overcome both the strong PCHC self-association and the large configurational entropy associated with the long PCHC sequences, consistent with the classical thermodynamic framework for hydrogen-bonded polymer blends.^59^ As a result, the PSHPMI segments are partially decoupled from cooperative segmental relaxation, leading to a *T*_g_ plateau. When the PSHPMI fraction increases to ≥70 wt %, the density of the imide-based hydrogen-bond donors and rigid aromatic units becomes sufficiently high, such that the interassociation between PCHC and PSHPMI becomes comparable to or exceeds the self-association within each block. Under these conditions, the two segments participate in more cooperative segmental motion, and the *T*_g_ shifts sharply upward, approaching Fox-type behavior. This transition reflects a crossover from a PCHC-dominated dynamic regime to a PSHPMI-dominated hydrogen-bonded network.

Hydrogen-bonding interactions can be elucidated through thermal analyses in combination with infrared spectroscopy, which is a versatile technique for investigating solid-state polymer interactions, offering both qualitative and quantitative analytical capabilities. Figure 5a displays the FTIR spectral regions, representing the OH stretching absorptions of pure PCHC, pure PSHPMI, and PCHC-*b*-PSHPMI copolymers with different PSHPMI compositions. The phenolic O–H stretching band of PSHPMI appeared in the range of 3100–3700 cm^–1^, comprising signals from free O–H groups at 3550 cm^–1^ and self-associated O–H···O–H/O-H···O=C groups at 3440 cm^–1^.

**Figure 5:**
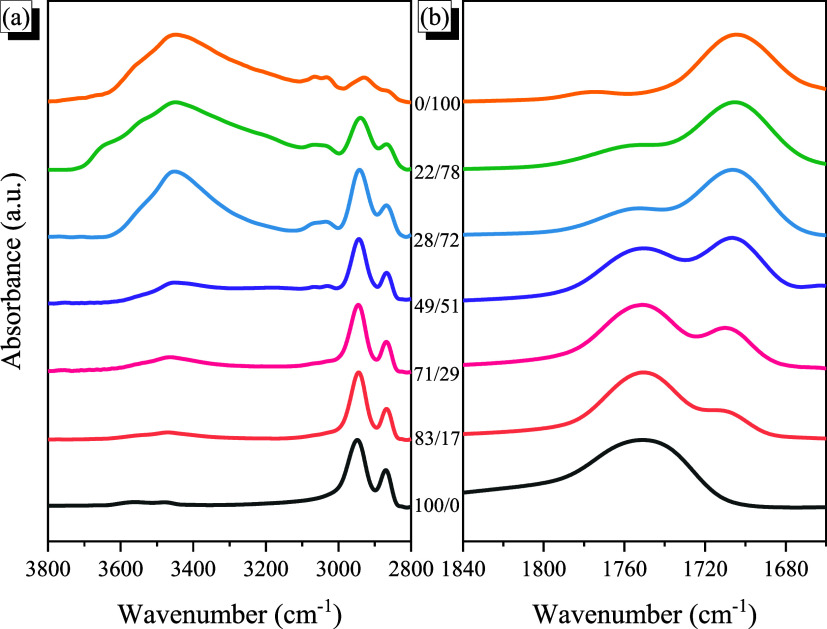
FTIR spectra of various PCHC-*b*-PSHPMI copolymers, recorded at 120 °C: (a) OH and (b) C=O stretching regions.

Upon increasing the concentration of PCHC, the absorption intensity of free O–H was decreased, and the signal for the self-associated OH units from the PSHPMI segment was shifted to a higher wavenumber (ca. 3470 cm^–1^), representing a transformation to relatively weak intermolecular hydrogen bonding in the PCHC-*b*-PSHPMI copolymers. Since the average H-bonding strength could be measured from the frequency difference (Δν) between the signals for the H-bonded and free OH units,^54^ thus the intermolecular H-bonding of the C=O units of PCHC with the OH units of PSHPMI (Δν = 80 cm^–1^) is weaker than the self-associated OH O–H···O–H/O-H···O=C of the pure PSPHMI segment (Δν = 110 cm^–1^), which is consistent with the negative *q* value based on the modified Kwei equation.

Figure 5b shows the corresponding C=O absorption of various PCHC-*b*-PSHPMI copolymers. We can expect several C=O absorption peaks for the PCHC-*b*-PSHPMI copolymers. For example, the PCHC segment possesses two absorptions at 1763 and 1735 cm^–1^, corresponding to free and intramolecular hydrogen-bonded C=O units, respectively.^12^ The PSHPMI copolymer segment shows two major signals for C=O units at 1775 and 1705 cm^–1^ due to the asymmetric and symmetric HPMI units, respectively.^40^ Furthermore, due to the self-association of O–H···O=C, hydrogen-bonding interactions were expected, and thus, the free C=O units of the symmetric HPMI units were observed at 1720 cm^–1^ based on two-dimensional (2D) FTIR analyses, which will be proved later. In addition, the interassociation hydrogen-bonding interaction between the C=O units of PCHC and phenolic OH units of the PSHPMI segment was also expected at ca. 1735 cm^–1^ for the PCHC-*b*-PSHPMI copolymers. First, the self-association of the O–H···O=C hydrogen-bonding interaction of the pure PSHPMI segment at 1720 cm^–1^ was gradually shifted to a relatively higher wavenumber, which was located at 1710 cm^–1^ for the PCHC-*b*-PSHPMI17 copolymer, indicating that the self-association of the O–H···O=C hydrogen-bonding interaction of the PSHPIM segment was transferred to an interassociation hydrogen-bonding interaction between the C=O units of the PCHC and phenolic OH units of the PSHPMI segment. Second, to simplify the quantitative analyses of these C=O units by curve fitting for these various PCHC-*b*-PSHPMI copolymers, we ignored the asymmetric C=O units at 1775 cm^–1^ because of the much smaller absorption compared to symmetric C=O, as displayed in the pure PSHPMI copolymer, and also combined the intramolecular hydrogen-bonded C=O units of the pure PCHC segment and the interassociation hydrogen-bonding interaction of the O–H···O=C units of the PCHC-*b*-PSHPMI copolymers since they both exhibited at ca. 1735 cm^–1^.

Figure 6A shows the representative curve-fitting results for the four peaks in the spectra of the various PCHC-*b*-PSHPMI copolymers. In these copolymers, the relative area fraction corresponding to the intermolecular hydrogen-bonded C=O units of the PCHC segment was increased with increasing PSHPMI composition, as shown in Figure 6B. The self-association of the O–H···O=C hydrogen-bonding interaction was decreased upon decreasing the PSHPMI composition (Figure 6C), as expected.

**Figure 6:**
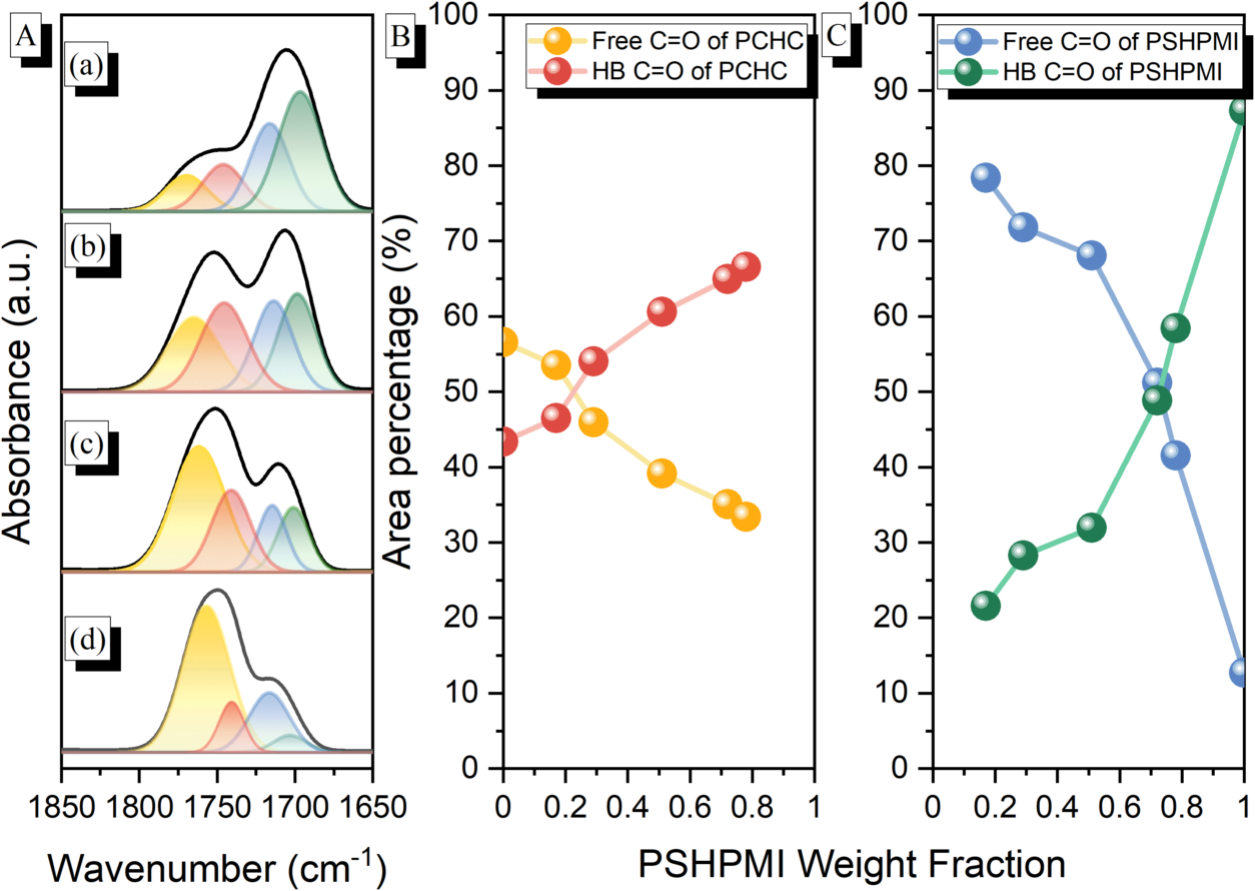
(A) Curve fitting of the C=O absorptions of selected (a) PCHC-*b*-PSHPMI78, (b) PCHC-*b*-PSHPMI72, (c) PCHC-*b*-PSHPMI51, and (d) PCHC-*b*-PSHPMI17. (B) Area fractions of the free C=O, intermolecular H-bonded C=O of the PCHC segment, and (C) free C=O and self-association H-bonded C=O of the PSHPMI segment.

In addition, we also investigated the corresponding various temperature FTIR analyses between the PCHC and PSHPMI segments to verify that hydrogen-bonding interactions occurred in the PCHC-*b*-PSHPMI copolymers, as shown in Figure 7A,B. These PCHC-*b*-PSHPMI copolymers undergo hydrogen-bonding interactions based on their chemical structures and thermal analytical data. One effective method for comprehending hydrogen bonding in polymeric materials is FTIR spectroscopy over a temperature range of 60–180 °C, and the hydrogen-bonded interactions were further analyzed by using 2D-FTIR spectra,^60^ which not only enhanced the spectral resolution but also allowed us to determine the relationship between free and hydrogen-bonded O–H and C=O units. The PCHC-*b*-PSHPMI51 copolymer was chosen for analysis and measurement, as shown in Figure 7A,B. Clearly, all the absorptions of the OH and C=O units were shifted to higher wavenumbers upon increasing the temperature, indicating that the inter/intramolecular hydrogen bonding would be destroyed with an increase of temperature, as expected.^54^ In Figure 7C,D, the correlation between the O–H and C=O vibrations is clearly observed. Each O–H and C=O unit comprises contributions from both free and intermolecular hydrogen-bonded species. The two-dimensional synchronous FTIR spectra, as shown in Figure 7C, resolve two distinct signals of O–H units at ca. 3550 and 3440 cm^–1^, corresponding to free and hydrogen-bonded OH units from the HPMI units and four C=O signals at ca. 1765, 1740, 1720, and 1700 cm^–1^, due to the free and hydrogen-bonded C=O units from the PCHC and HPMI segments, as mentioned previously. Conspicuously, two positive cross peaks appeared below the frame (3550 vs 1765 cm^–1^, 3500 vs 1720 cm^–1^), indicating that the free OH and free C=O of the carbonate/anhydride exhibited the same change, as the temperature increased.^55^ However, two negative peaks appeared (3550 vs 1740 cm^–1^, 3550 vs 1700 cm^–1^) for free OH with hydrogen-bonded C=O units, indicating that these two absorption bands varied in opposite directions, as expected. The asynchronous 2D-FITR spectra are summarized in Figure 7D, and the sequential order is 3550 > 3440 > 1763 > 1740 > 1720 > 1700 cm^–1^, indicating that the free OH from PSHPMI was the most sensitive and the self-association of the O–H···O=C hydrogen-bonding interaction of the PSHPMI segment was less sensitive upon increasing the temperature, which provided high thermal stability.

**Figure 7:**
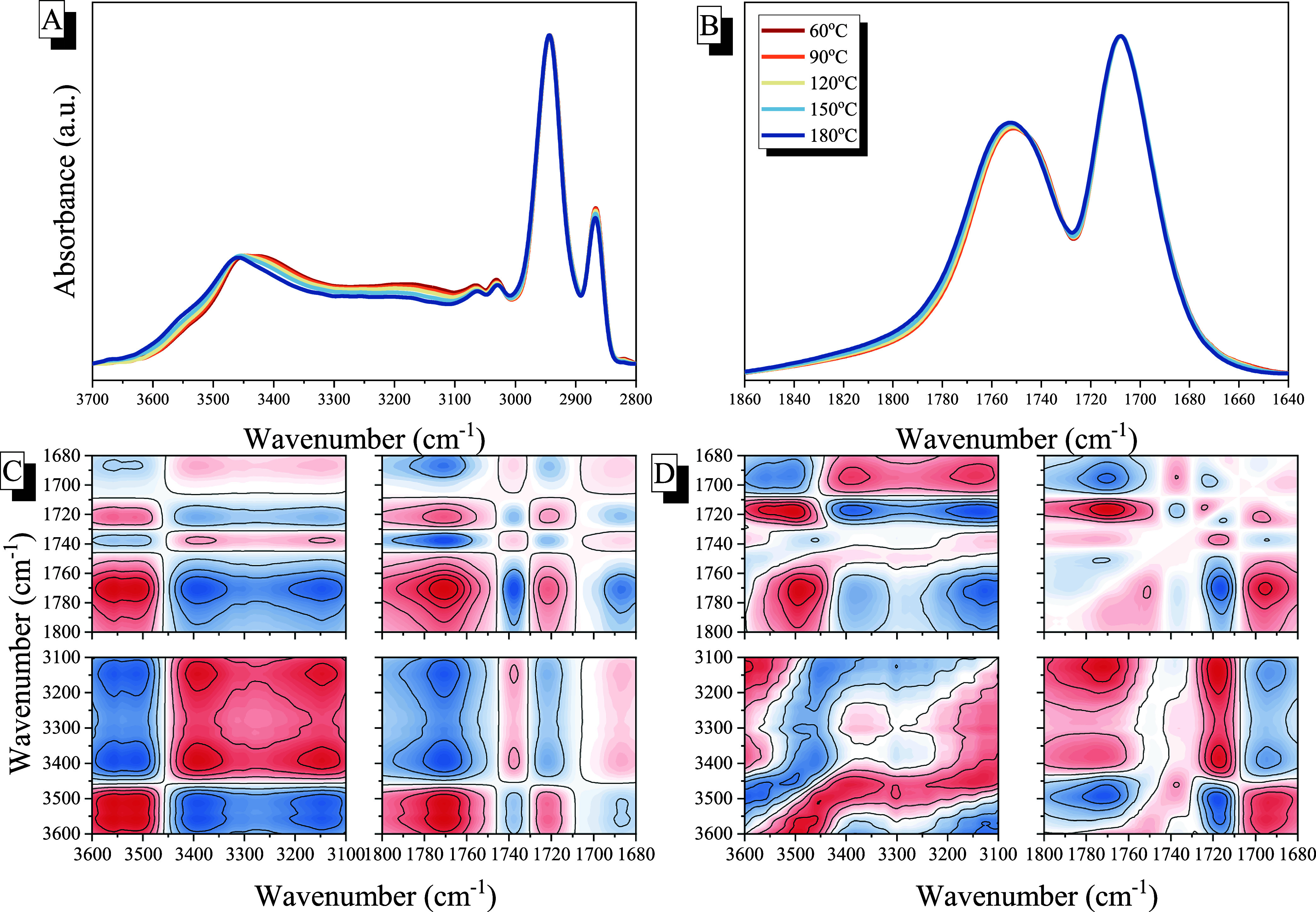
FTIR spectra of the C=O and OH regions, recorded from 60 to 180 °C, for the PCHC-*b*-PSHPMI51 copolymer. (A) OH and (B) C=O absorptions, and their corresponding 2D-FTIR spectra of (C) synchronous and (D) asynchronous correlation maps.

Solid-state NMR spectroscopy also provided insights into the intermolecular hydrogen-bonding interactions, domain sizes, and molecular mobility in the PCHC-*b*-PSHPMI copolymers, as shown in Figures 8 and 9. Figure 8a displays the ^13^C solid-state CP/MAS NMR spectra of various PCHC-*b*-PSHPMI copolymers, and the corresponding peaks were assigned as the same as Figure S2. Three important signals were observed at 179.04 ppm for C=O units and 157.80 ppm for C–OH units from the PSHPMI segment and 154.19 ppm for C=O units from the PCHC segment. Figure 8b shows the chemical shifts of these three signals of various PCHC-*b*-PSHPMI copolymers, where the chemical shift of the C=O units from the pure PCHC segment (154.19 ppm) was shifted downfield upon increasing PSHMPI composition and was observed at 155.21 ppm for the PCHC-*b*-PSHPMI78 copolymer. Furthermore, the C–OH units from the PSHPMI segment also exhibited a downfield chemical shift upon increasing PCHC composition from 157.80 to 158.59 ppm for the PCHC-*b*-PSHPMI17 copolymer. However, the C=O units from the PSHPMI segment were observed upfield upon increasing the PCHC compositions from 179.04 to 178.11 ppm, also indicating that the self-association of the O–H···O=C hydrogen-bonding strength of PSHPMI was decreased upon increasing the PCHC compositions in PCHC-*b*-PSHPMI copolymers, which is consistent with the DSC and FTIR analyses. In addition, Figure 8c,d display the signals of the three carbon nuclei of pure PCHC, pure PVPh, and various PCHC-*b*-PSHPMI copolymers. Combining experimental data (solid line) with simulated data (dashed line)^56^ can also confirm the possible hydrogen-bonding interactions in various PCHC-*b*-PSHPMI copolymers. The simulated spectra for each copolymer were generated by directly summing the experimental ^13^C solid-state NMR spectra of pure PCHC and pure PSHPMI in their corresponding compositions. The experimental spectra of various PCHC-*b*-PSHPMI copolymers differed substantially from the simulated spectra. The experimental spectra displayed broad and complex signals, indicative of intermolecular hydrogen-bonding interactions between the C=O units of the PCHC and PSHPMI segments and the phenolic O–H groups of the PSHPMI segment. Moreover, the C=O units of PSHPMI shifted upfield with increasing PCHC content in the copolymers, thereby confirming the presence of specific interactions between these two segments (Figure 8c). A resonance signal was also observed between 154 and 157 ppm, as shown in Figure 8d, and relative to the simulated spectra, the experimental data exhibited a pronounced downfield shift. This observation is consistent with the notion that the phenolic O–H units in PSHPMI interact not only with the C=O groups of PCHC but also with the imide C=O units in PSHPMI via self-association.

**Figure 8:**
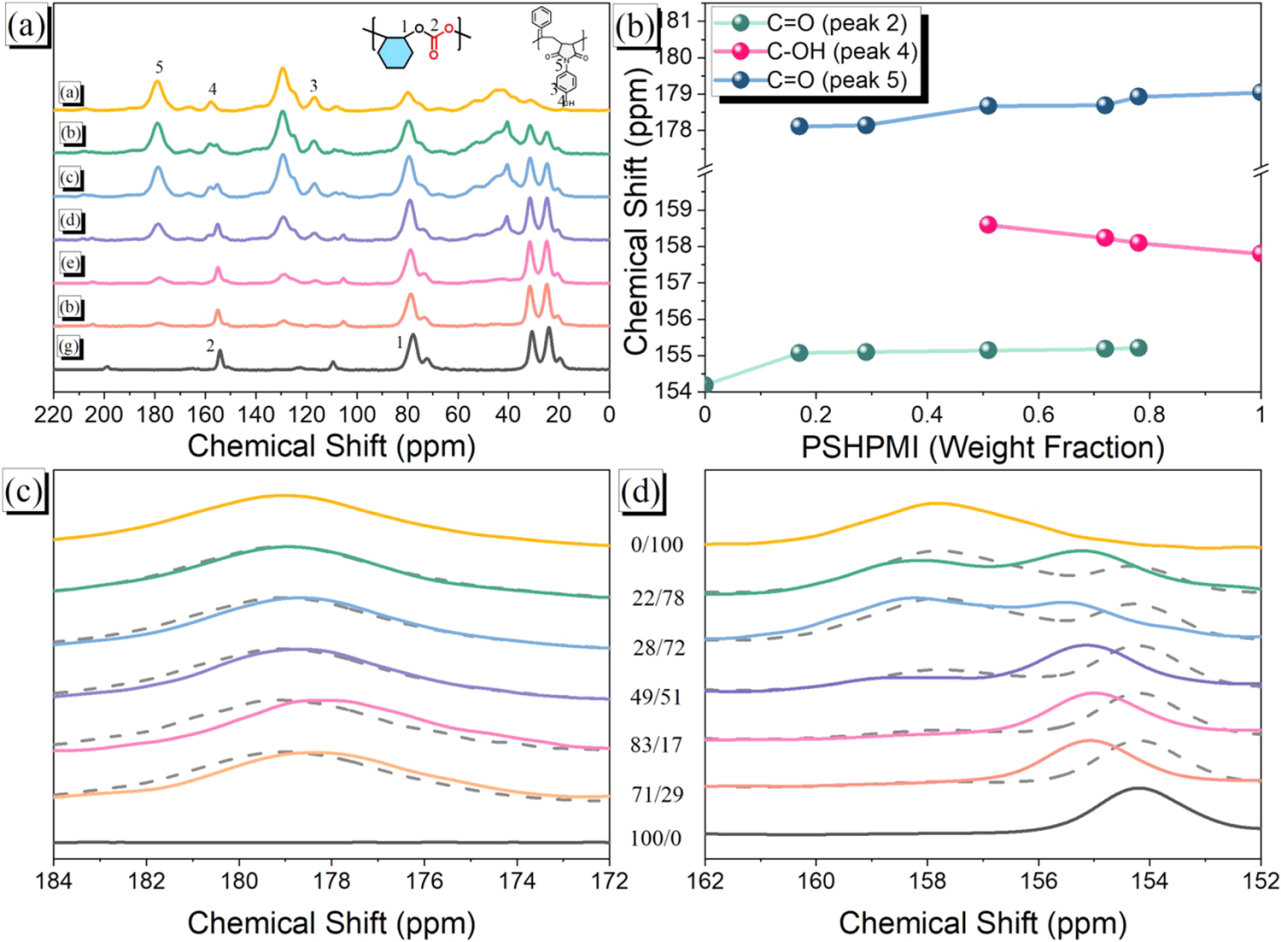
(a) High-resolution solid-state ^13^C NMR spectroscopy was performed at 25 °C for various PCHC-*b*-PSHPMI copolymers. (b) Chemical shifts of PCHC-*b*-PSHPMIs with various PSHPMI contents. Selected carbon nuclei in solid-state ^13^C NMR spectra: (c) C–OH and C=O (PCHC segment), and (d) C=O (PSHPMI segment) of various PCHC-*b*-PSHPMI copolymers.

**Figure 9:**
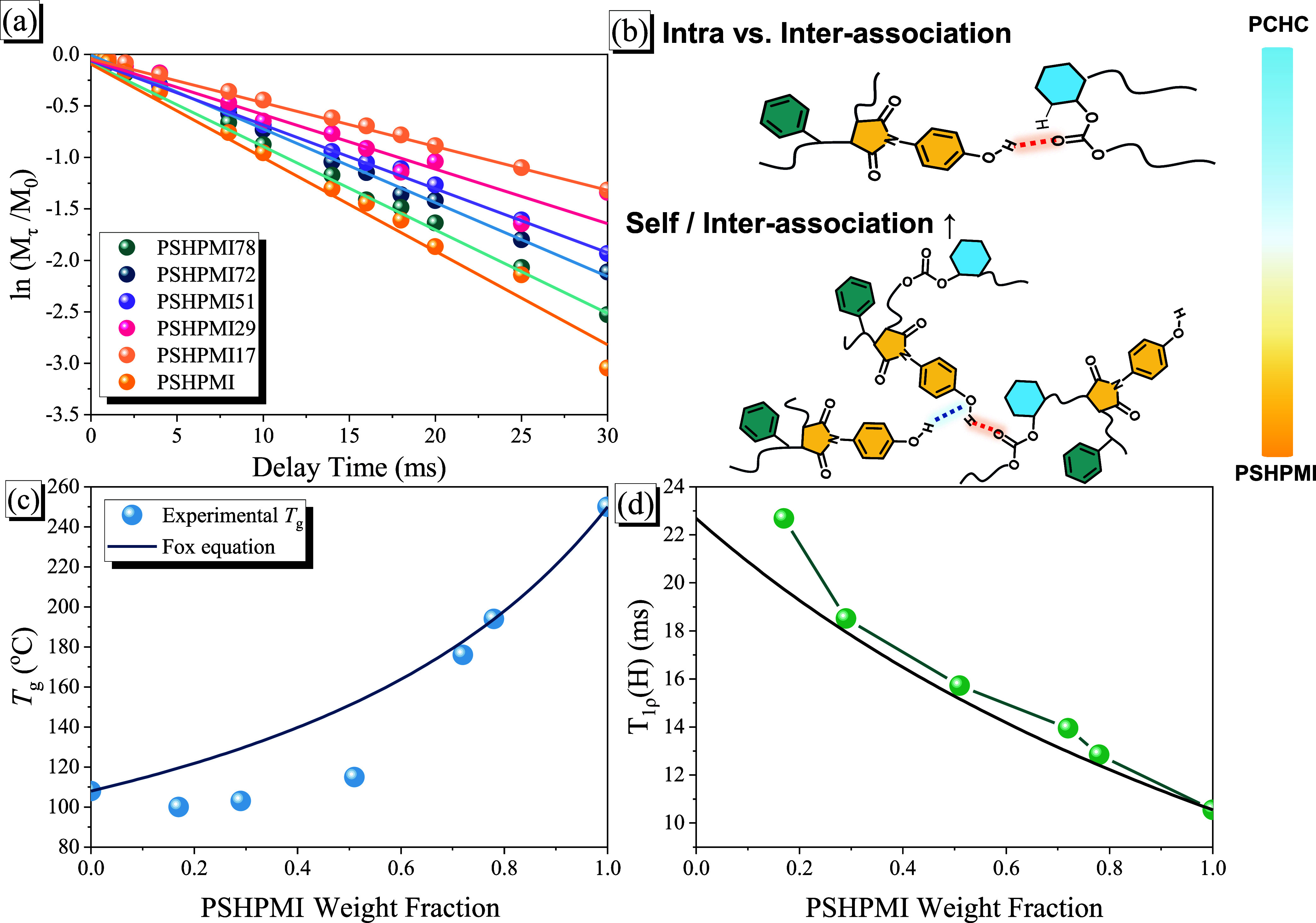
(a) Semi-logarithmic plots of the magnetization intensities of the signals at 155 ppm, plotted with respect to the delay time of various PCHC-*b*-PSHPMIs; (b) possible H-bonding interactions of PCHC-*b*-PSHMPI copolymers with various PSHPMI compositions, (c) Fox equation, and (d) plots of *T*_1ρ_(H) calculated with respect to the PSHPMI composition of the PCHC-*b*-PSHPMI copolymers.

The domain size and molecular mobility in hydrogen-bonded blends or copolymers can be evaluated from the proton spin–lattice relaxation time in the rotating frame, *T*_1ρ_(H).^28^ In this method, *T*_1ρ_(H) is determined by measuring the decay of the NMR signal intensity under a spin-lock field. The relationship is expressed as $M_{\tau }=M_{0}\exp\left(-\frac{\tau }{T_{1\rho }\left(H\right)}\right)$ , where τ is the spin-locking time, and *M*_0_ and *M*_τ_ are the signal intensities measured at the beginning of spin-locking and after τ seconds, respectively. Figure 9a shows the plots of ln­(*M*_0_/*M*_τ_) with respect to τ for the C=O units at δ = 155 ppm for all copolymer compositions, where the experimental data provided a single exponential decay function. Using the one-dimensional diffusion equation and the average diffusive route length, we determined a single value of *T*_1ρ_(H) in the copolymer. This result showed that the copolymer miscibility dimensions were less than 2–3 nm. When two polymers are uniformly mixed, the glass transition temperature (*T*_g_) of the resulting blend typically falls between the *T*_g_ values of the individual components. The Fox equation is commonly employed to predict *T*_g_ of such miscible polymer blends or copolymers based on the weight fractions of each component, expressed as $\frac{1}{T_{g}}=\frac{W_{1}}{T_{g1}}+\frac{W_{2}}{T_{g2}}$ , where *T*_g_ is the glass transition temperature of the block copolymer, *T*_g1_ and *T*_g2_ are the glass transition temperatures of the pure polymers, and *W*_1_ and *W*_2_ represent their respective weight fractions. The *T*_g_ values of the block copolymers, as shown in Figure 9c, deviate from the value predicted by the Fox equation negatively, suggesting the presence of weaker intermolecular hydrogen-bonding interactions between the PCHC and PSHPMI segments in these block copolymers, as discussed previously.^54^ Furthermore, Figure S3 shows the DSC thermal analyses of PSHPMI/PCHC binary blends; the two *T*_g_ values were observed, indicating that this binary blend is immiscible because of its weaker intermolecular hydrogen-bonding interaction between the PCHC and PSHPMI segments. As a result, the short-range attractive interaction from the covalent bonded linkage between the PCHC and PSHPMI segments of the PCHC-*b*-PSHPMI copolymers could improve the miscibility behavior significantly in this case.^54^ Figure 9d shows the values of *T*_1ρ_(H) with respect to the PSHPMI weight fraction, and the single *T*_1ρ_(H) value of the PCHC-*b*-PSHPMI copolymers can also be regarded as a single-phase material and is considered thermodynamically miscible within this hydrogen-bonded copolymer system. The experimental relaxation rates for each copolymer composition exhibited positive deviations from the values calculated using eq 2,^28,61,62^ suggesting that the free volume and density of the copolymers differ substantially from the predicted values

2 $$\frac{1}{T_{1\rho }\left(H\right)}=\frac{N_{A}M_{A}}{N_{T}}\left(\frac{1}{T_{1\rho }\left(H_{A}\right)}\right)+\frac{N_{B}M_{B}}{N_{T}}\left(\frac{1}{T_{1\rho }\left(H_{B}\right)}\right)$$

where *A* and *B* denote the respective segments of the copolymer; *M_i_* is the mole fraction of component *i*; *N_i_* is the number of protons in component *i*; and *T*_1ρ_(*H_A_*) and *T*_1ρ_(*H_B_*) represent the proton spin–lattice relaxation times in the rotating frame for components *A* and *B*, respectively. This equation is ideal close to the Fox equation, and a positive deviation from the predicted curve was observed, indicating that the domain size was larger than the predicted value because of the weaker intermolecular hydrogen-bonding strength between the PCHC and PSHPMI segments. The loose and chain-expanded structure was expected because of the larger *T*_1ρ_(H) value with a larger domain size; thus, the thermal properties could not be enhanced significantly upon increasing the PSHPMI compositions, and even exhibited a negative deviation based on both the Fox and Kwei equations. The possible inter- and self-association of hydrogen-bonding interactions is summarized in Figure 9b for various PCHC-*b*-PSHPMI copolymers. At lower PSHPMI compositions (<51 wt %), the weaker intermolecular hydrogen bonding between the C=O units of PCHC and phenolic OH units of the PSHPMI segments was dominant, and thus, the *T*_g_ value was similar to that of the pure PCHC segment (*T*_g_ = 103–115 °C), which was much lower than the value predicted by the Fox equation. However, at relatively higher PSHPMI compositions (72 and 78 wt %), the stronger self-association of the O–H···O=C hydrogen-bonding strength of the PSHPMI segment was dominant, and thus, the *T*_g_ value (*T*_g_ = 176–194 °C) could be enhanced and predicted by the Fox equation. Overall, the *T*_g_ value of the PCHC segment could be increased only for block copolymers of PCHC-*b*-PSHPMI through covalent bonding, and also needs to be achieved at relatively higher PSHPMI compositions in block copolymers. As a result, enhancing the thermal properties of CO_2_-based copolymers remains challenging because of the strong intramolecular hydrogen-bonding interactions of the carbonate segments. The possible design should involve weaker self-association hydrogen-bonding interactions and random or alternating sequence distributions of hydrogen-bonded donor segments within the CO_2_-based copolymers.

## Conclusions

We successfully synthesized various CO_2_-based PCHC-*b*-PSHPMI block copolymers through the combination of CO_2_/CHO ring-opening and RAFT copolymerizations, which was confirmed by FTIR, NMR, and GPC analyses. Thermal analyses revealed that the introduction of the PSHPMI block segment significantly enhanced the *T*_g_ behavior at relatively higher PSHMPI compositions (>72 wt %), indicating the balance of self-association and interassociation hydrogen-bonding interactions of the C=O units of the PCHC and PSHMPI segments within the phenolic OH units from the PSHMPI segments, which could be confirmed by FTIR and solid-state NMR analyses. Overall, this work demonstrates an effective strategy to design CO_2_-based functional block copolymers with tunable thermal properties through the combination of ring-opening and RAFT copolymerizations. The synthetic approach and structural tunability established in this study may open new avenues for developing advanced CO_2_-based block copolymers for potential applications in sustainable materials, solid electrolytes, and subnanometer patterns.

## Supplementary Material



## Notes

Characterization methods; synthesis and analyses of LZn_2_(OAc)_2_, DDMAT, and PSHPMI; DSC analyses of PCHC/PSHPMI blends; ^13^C NMR spectra of the PCHC-*b*-PSHPMI copolymer (PDF)
