# Supplementary material for: Highly Thermally Stable and Miscible CO2‑Based Block Copolymers by the Combination of Ring-Opening and RAFT Copolymerizations through Mediated Hydrogen Bonding Interactions
Source: Macromolecules. 2026 Jan 26;59(3):1346–57. doi: 10.1021/acs.macromol.5c03069 (PMC12895532; doi:10.1021/acs.macromol.5c03069)
Supplement: Supplementary file 1 [file ma5c03069_si_001.pdf]

Supporting Information

Highly Thermal Stable and Miscible CO_2-Based Block Copolymers by the Combination of Ring-Opening and RAFT Copolymerization through Mediated Hydrogen Bonding Interactions

Yen-Ling Kuan, Yu-Chun Chiu, Yun-Sheng Ye and Shiao-Wei Kuo*

Department of Materials and Optoelectronic Science, Center for Functional Polymers and Supramolecular Materials, National Sun Yat-Sen University, Kaohsiung 80424, Taiwan

To whom should be corresponding

E-mail: kuosw@faculty.nsysu.edu.tw

Characterization

^1H and ^{13}C NMR spectra were recorded using a JEOL ECZ600R NMR spectrometer, with $CDCl_3$ or DMSO as an external standard. Weight-average (M_w) and number-average (M_n) molecular weights and polydispersity indexes (M_w/M_n) were determined using a Waters 510 gel permeation chromatograph. IR spectra were recorded using a Bruker Tensor 27 FTIR spectrophotometer by the conventional KBr disk method; 32 scans were collected at a spectral resolution of 4 cm^{-1}. A temperature-controlled compartment holder was used to record FTIR spectra at various temperatures, thereby maintaining dry sample films. Glass transition temperatures were measured using a TA Q20 differential scanning calorimeter. Approximately 5 mg of sample was placed on the DSC sample pan and heated from -90 to 280 °C at a heating rate of 10 °C min^{-1} under a N_2 atmosphere (50 mL min^{-1}). Thermogravimetric analysis (TGA) was performed using a TA Q50 instrument. Approximately 10 mg of sample was placed on a TGA crucible and set onto a platinum pan, then heated from 40 to 800 °C at a heating rate of 20 °C min^{-1} under a N_2 atmosphere (balance purge flow: 40 mL min^{-1}, sample purge flow: 60 mL min^{-1}). High-resolution solid-state ^{13}C NMR spectroscopy was performed at 25 °C using a Bruker AVANCE III spectrometer operated at a resonance frequency of 100.62 MHz. The experiments were performed using the cross-polarization (CP)/magic-angle spinning (MAS)/high-power dipolar decoupling (DD) technique. The proton spin-lattice relaxation time in the rotating frame ($T_{1\rho}H$) was determined through carbon observation using a 90° τ-spin lock pulse sequence prior to CP. The acquisition was performed at delay times (s) ranging from 0.1 to 30 ms with a contact time of 1.0 ms.

Ligand synthesis (LH)

Dissolve 2,2-dimethyl-1,3-propanediamine in methanol and drop it into the *o*-vanillin in the methanol. The mixture was stirred with a magnetic stirrer at room temperature for 4 hours, and the solvent was removed to obtain a yellow powder. The synthesis method was reported by Williams' group [1]. FTIR (KBr, cm^{-1}): 1632 (aromatic C=C), 3450 (O–H); ^1H NMR (500 MHz, $CDCl_3$, δ, ppm): 1.07 (s, 6H, $CC\mathbf{H}_3$), 3.49 (s, 4H, $NC\mathbf{H}_2$), 3.91 (s, 6H, $OC\mathbf{H}_3$), 6.77-6.95(m, 6H, Ar**H**), 8.32 (s, 2H, N=C**H**), 14.15 (br, 2H, CO**H**); ^{13}C NMR (125 MHz, $CDCl_3$, δ, ppm): 24.3 ($C\mathbf{C}H_3$), 36.3 ($\mathbf{C}$-CH_3), 56.2 ($O\mathbf{C}H_3$), 67.5 ($N\mathbf{C}H_2$), 114.3-123.5 (Ar**C**), 149.1 ($\mathbf{C}OCH_3$), 152.8 (**C**OH), 166.6 (N=**C**).

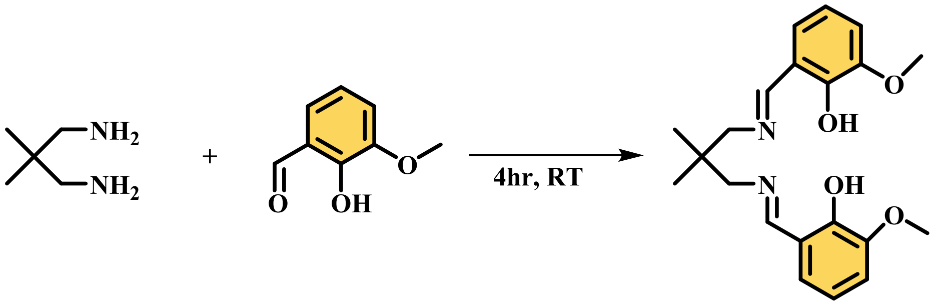

diamine ***o*-vanillin** **LH**

Scheme S1. The synthesis of LH.

Catalyst synthesis ($LZn_2(OAc)_2$)

Dissolve $Zn(OAc)_2{\cdot}2(H_2O)$ in methanol and drop it into the prepared LH and stir overnight. After the removal of solvent in vacuo. The pure product was obtained after washing with hexane, followed by crystallization from THF/hexane. The synthesis has been reported by Williams' group [1]. FTIR (KBr, cm^{-1}): 1624 (aromatic C=C); ^1H NMR (500 MHz, $CDCl_3$, δ, ppm): 1.03 (s, 6H, $CC\mathbf{H}_3$), 1.97 (s, $C(O)C\mathbf{H}_3$, 6H), 3.74 (s, 4H, $NC\mathbf{H}_2$), 3.86 (s, 6H, $OC\mathbf{H}_3$), 6.50-6.90 (m, 6H, Ar**H**), 8.04 (s, 2H, N=C**H**); ^{13}C NMR (125 MHz, $CDCl_3$, δ, ppm): 23.3($C(O)\mathbf{C}H_3$), 25.4 (C-$\mathbf{C}H_3$), 35.5 ($\mathbf{C}CH_3$), 56.0 ($O\mathbf{C}H_3$), 75.0 ($N\mathbf{C}H_2$), 114.2-118.6(Ar**C**), 127 ($\mathbf{C}OCH_3$), 150.9 (**C**-OH), 171.1 (N=**C**).

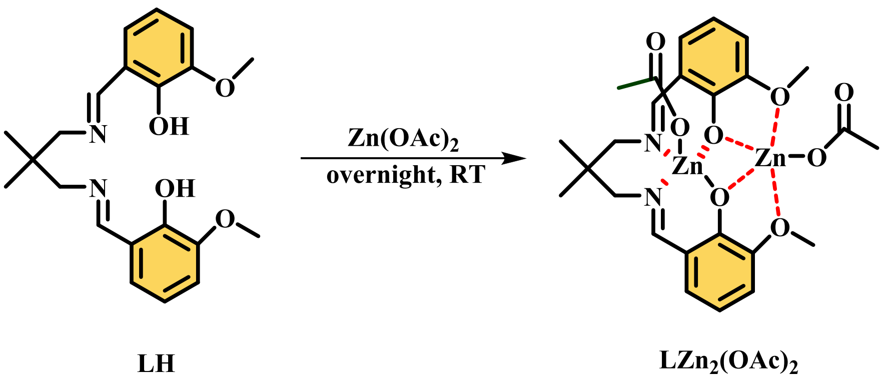

Scheme S2. The synthesis of $LZn_2(OAc)_2$.

Synthesis of *s*-dodecyl-*s'*-(α,α′-dimethyl-α″-acetic acid) trithiocarbonate (DDMAT)

A mixture of 1-dodecanethiol (10.12 g, 0.05 mol) and Aliquat 336 (0.914 mL, 0.002 mol) in acetone (30 mL) was placed in a 250 mL round-bottom flask, cooled to 0 °C under a nitrogen atmosphere, and stirred. A 50 wt% aqueous sodium hydroxide solution (2.1 g, 0.0525 mol) was added dropwise over 20 min, after which the mixture was stirred for an additional 20 min. Carbon disulfide (3.805 g, 0.05 mol) dissolved in acetone (6.4 mL) was then added dropwise over 20 min, followed by stirring for 10 min. Chloroform (6 mL, 0.075 mol) was introduced, and a sodium hydroxide solution (10 g, 0.25 mol) was added dropwise over 30 min. The reaction mixture was stirred overnight at ambient temperature. Deionized water (75 mL) was added, followed by concentrated hydrochloric acid (12.5 mL). The nitrogen stream was removed, and acetone was evaporated under reduced pressure. The precipitated solid was collected by filtration, stirred in isopropanol (125 mL), filtered as before, and the filtrate was concentrated by rotary evaporation. The resulting crude solid was purified by recrystallization from hexane. ^{1}H NMR (500 MHz, $CDCl_3$, δ, ppm): 0.89 (t, 3H, -CH_2**CH_3**), 1.26–1.47 (m, 18H, -C**H_2**C**H_2**-), 1.68 (m, 2H, -C**H_2**CH_2S-), 1.73 (s, 6H, -C-C**H_3**), 3.29 (t, 2H, -C**H_2**S-); ^{13}C NMR (125 MHz, $CDCl_3$, δ, ppm): 177.56 (-**C**-O-), 55.37 (-S**C**-), 37.24 (1C, -**C**H_2S-), 31.76 (-**C**$H_2$$CH_2$S-), 29.43 (-**C**$H_2$$CH_2$-), 28.95 (-**C**$H_2$**C**$H_2$-), 27.79 (-**C**$H_2$$CH_2$-), 25.22 (-SC(**C**$H_3$)$_2$), 22.67 ($CH_3$**C**$H_2$-), 14.10 (-$CH_2$**C**$H_3$).

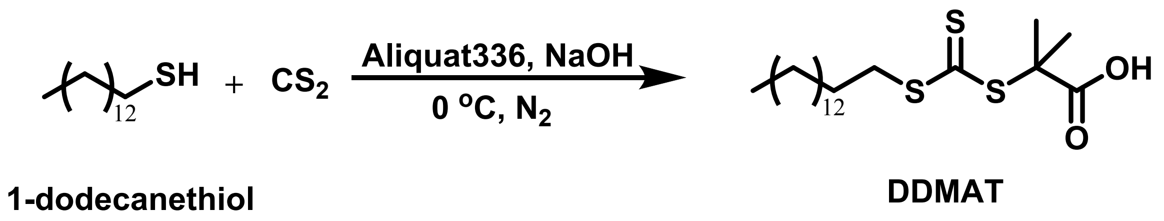

Scheme S3. The synthesis of DDMAT.

Copolymerization of P(S-*alt*-HPMI) (PSHPMI)

A solution of HPMI (2.08 g, 5.50 mmol), styrene (1.15 g, 5.50 mmol), and AIBN (5 wt%) in dry THF (15 mL) was prepared in a round-bottom flask and degassed through three freeze–pump–thaw cycles. Then heated for 24 hours at 80 °C, cooled to room temperature, and reprecipitated with methanol. FTIR (KBr, cm^{-1}): 1703 (C=O), 3482 (O–H); ^{1}H NMR (600 MHz, DMSO-d_6, δ, ppm): 6.83 (m, 2H, Ar**H**), 7.08 (m, 2H, Ar**H**), 7.11 (s, 2H, C**H**=C**H**), 9.72 (s, 1H, O**H**); ^{13}C NMR (125 MHz, DMSO-d_6, δ, ppm): 115.46, 122.54, 128.46 (Ar**C**), 134.56 (**C**H=**C**H), 157.06 (**C**-OH), 170.36 (**C**=O).

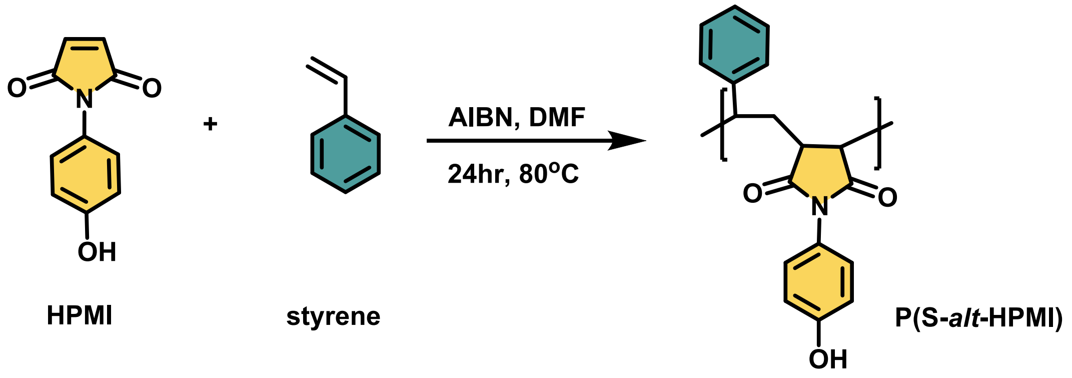

Scheme S4. The synthesis of P(S-*alt*-HPMI).

Blends of PCHC/PSHPMI

Various binary PCHC/PSHPMI blends were obtained via solution blending. The mixtures (5 wt%) in THF were stirred for 24 hours, and the solvent was then slowly removed over 3 days at 40 °C.

Table S1. Ring-opening polymerization of CHO/DDMAT/CO_2 catalyzed by $LZn_2(OAc)_2$[a].

**polymer**	**conv.[b] (CHO%)**	**TON[c]**	**TOF (h^{-1})[d]**	**% select.[e]**
PCHC-DDMAT	65	384.8	19.2	98

[a] Polymerization conditions: [CHO]:[DDMAT]:[cat.] = 1000:10:1. [b] Determined by ^{1}H NMR analysis of the crude product. [c] Turnover number (TON) = polymer (g)/catalyst (g). [d] Turnover frequency (TOF) = TON/time (hours). [e] Selectivity towards polymer over cyclohexene carbonate calculated from ^{1}H NMR spectrum.

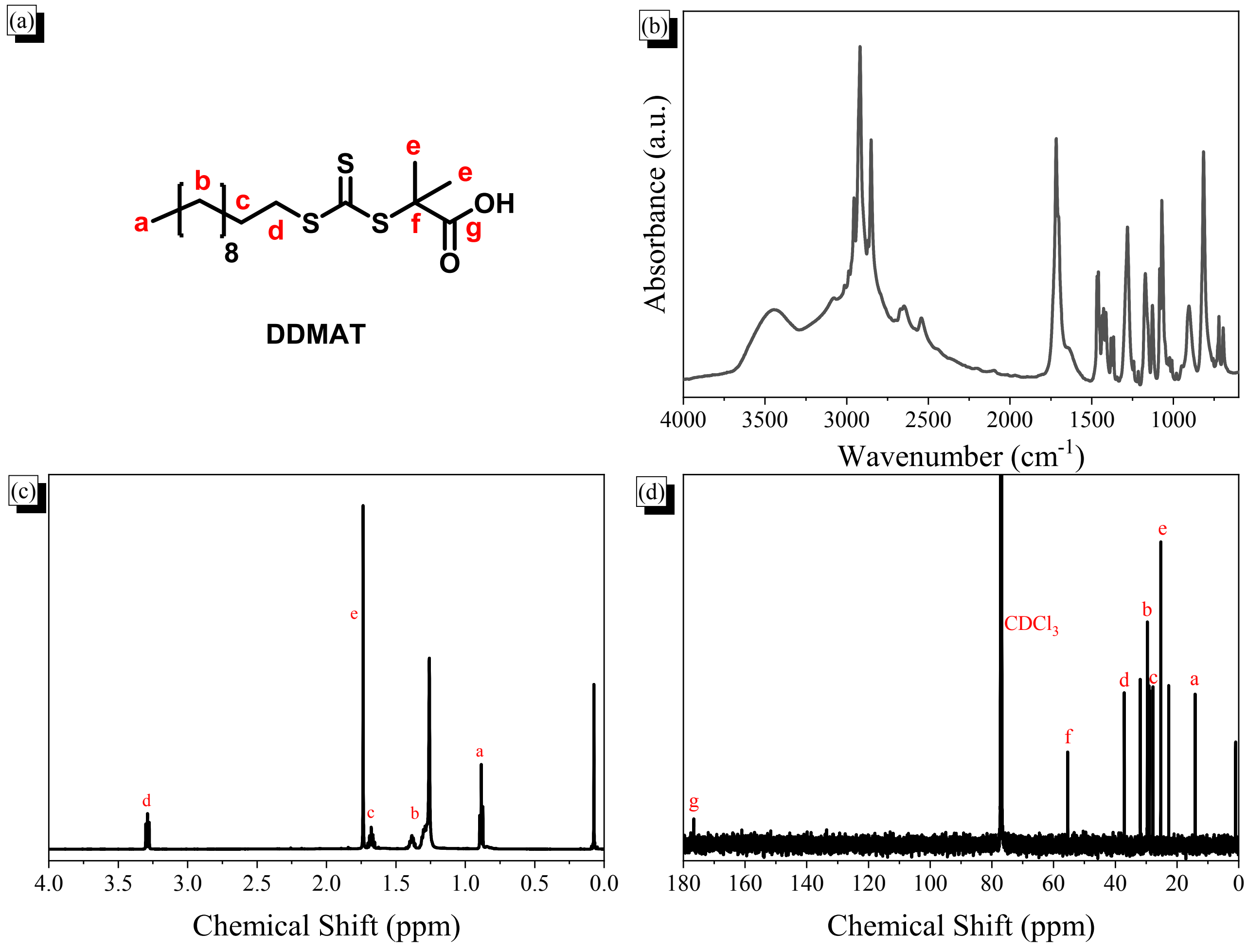

Figure S1. (a) Chemical structure of DDMAT, and its corresponding (b) FTIR, (c) ^{1}H NMR, and (d) ^{13}C NMR spectra.

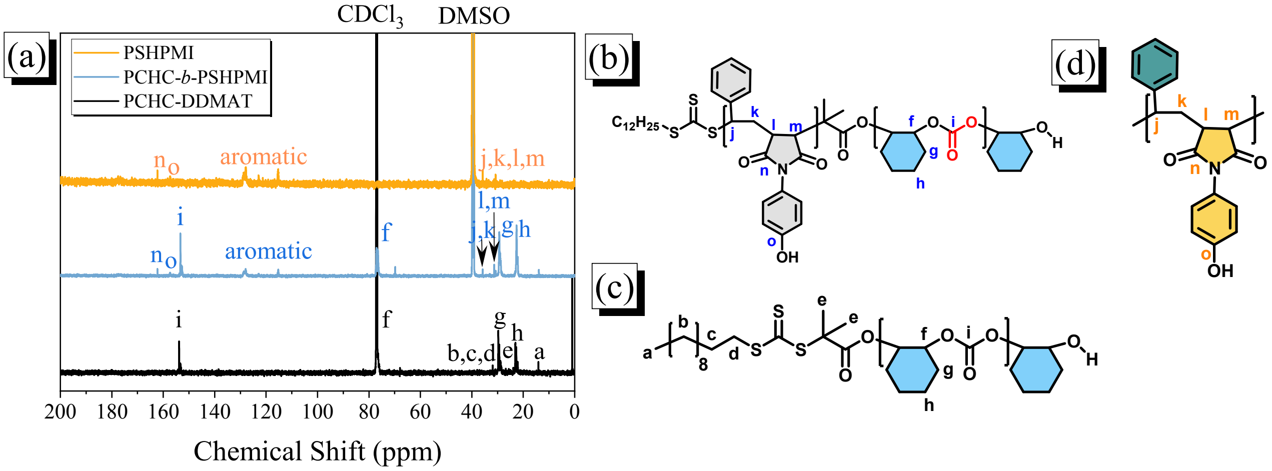

Figure S2. (a) ^{13}C NMR spectra of PCHC-DDMAT, PCHC-*b*-PSHPMI copolymer, and PSHPMI copolymer; (b, c, d) chemical structure and peak assignment: (b) PCHC-*b*-PSHPMI copolymer, (c) PCHC-DDMAT copolymer, and (d) PSHPMI copolymer.

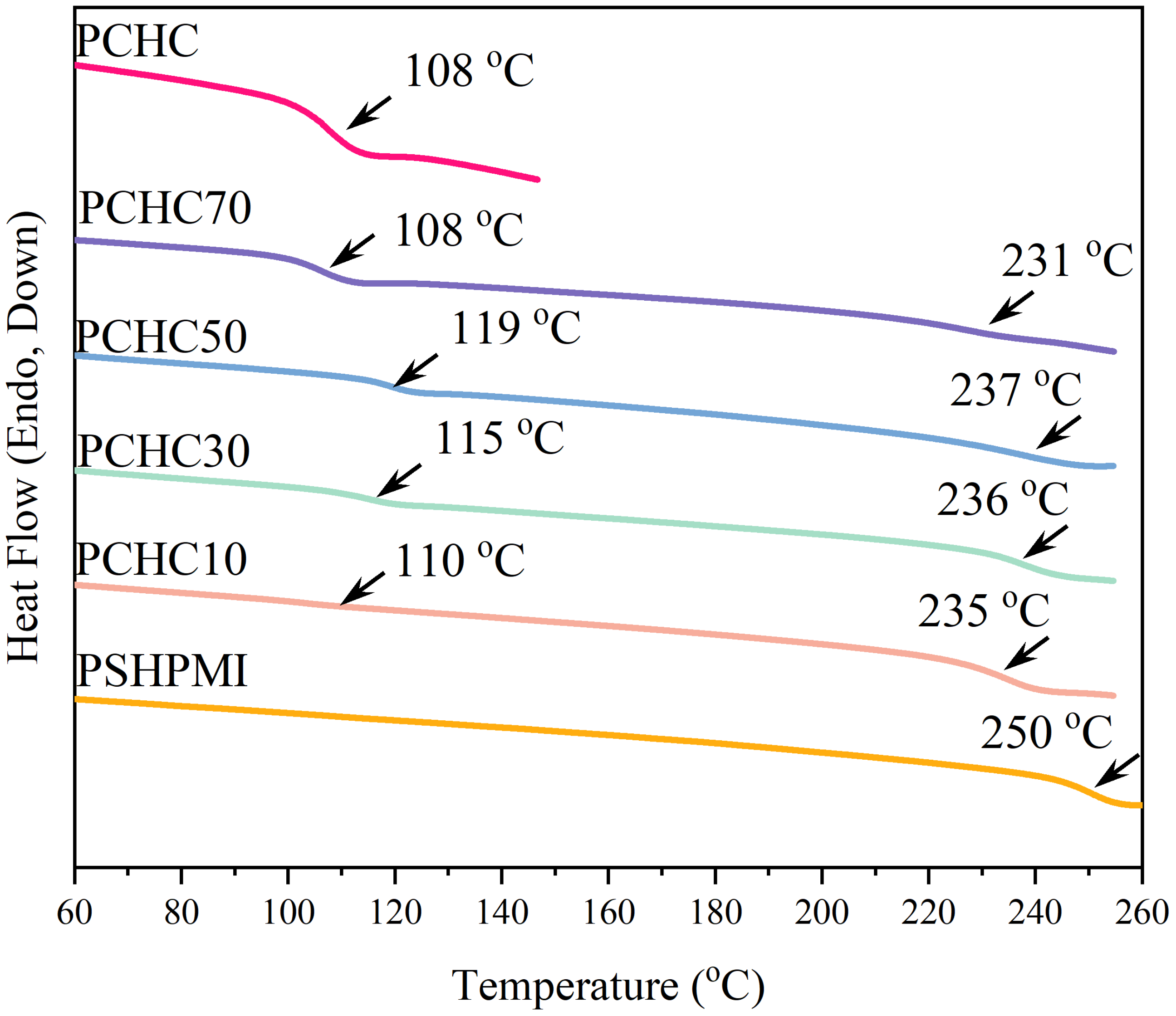

Figure S3. DSC thermal analyses of PCHC/PSHPMI binary blends.

References

[1] Thevenon, A.; Garden, J. A.; White, A. J. P.; Williams, C. K. Dinuclear zinc salen catalysts for the ring opening copolymerization of epoxides and carbon dioxide or anhydrides. *Inorg. Chem.* **2015**, *54*, 11906–11915, DOI: 10.1021/acs.inorgchem.5b02233
